# Community participatory learning and action cycle groups to reduce type 2 diabetes in Bangladesh (D:Clare): an updated study protocol for a parallel arm cluster randomised controlled trial

**DOI:** 10.1186/s13063-023-07243-x

**Published:** 2023-03-23

**Authors:** Carina King, Malini Pires, Naveed Ahmed, Kohenour Akter, Abdul Kuddus, Andrew Copas, Hassan Haghparast-Bidgoli, Joanna Morrison, Tasmin Nahar, Sanjit Kumer Shaha, AKAzad Khan, Kishwar Azad, Edward Fottrell

**Affiliations:** 1 Institute for Global Health, University College London, 30 Guildford Street, London, WC1N 1EH UK; 2 Department of Global Public Health, Karolinska Institutet, Stockholm, Sweden; 3 Diabetic Association of Bangladesh, Dhaka, Bangladesh

**Keywords:** Diabetes, Non-communicable diseases, Bangladesh, Participatory learning and action, Stepped-wedge trial, Cluster RCT

## Abstract

The “Diabetes: Community-led Awareness, Response and Evaluation” (D:Clare) trial aims to scale up and replicate an evidence-based participatory learning and action cycle intervention in Bangladesh, to inform policy on population-level T2DM prevention and control.

The trial was originally designed as a stepped-wedge cluster randomised controlled trial, with the interventions running from March 2020 to September 2022. Twelve clusters were randomly allocated (1:1) to implement the intervention at months 1 or 12 in two steps, and evaluated through three cross-sectional surveys at months 1, 12 and 24. However, due to the COVID-19 pandemic, we suspended project activities on the 20th of March 2020. As a result of the changed risk landscape and the delays introduced by the COVID-19 pandemic, we changed from the stepped-wedge design to a wait-list parallel arm cluster RCT (cRCT) with baseline data. We had four key reasons for eventually agreeing to change designs: equipoise, temporal bias in exposure and outcomes, loss of power and time and funding considerations.

**Trial registration** ISRCTN42219712. Registered on 31 October 2019.

The D:Clare trial (Diabetes: Community-Led Awareness, Response and Evaluation) was designed as a cluster randomised stepped-wedge trial, in Alfadanga Upazilla, Faridpur District, Bangladesh (ISRCTN42219712) [1]. The trial aims to evaluate the impact of a scaled-up community-based participatory learning and action (PLA) cycle intervention to prevent type 2 diabetes (T2DM) in a population of 120,000 people. The study began in January 2020, with a public consent and randomisation ceremony including community and Ministry of Health and Family Welfare representatives, on the grounds that all communities in the Upazilla would eventually receive the intervention in line with the stepped-wedge approach.

Bangladesh reported its first confirmed cases of SARS-CoV-2 on the 8th of March 2020. Due to concerns about infection risk to both staff and communities, we made a decision to suspend all field-based project activities on the 20th of March 2020 (Fig. 1). Early in the pandemic, evidence emerged that uncontrolled hyperglycaemia and T2DM were risks for severe COVID-19 infections and mortality, alongside older age, obesity and heart disease [2–4]. Given the focus and nature of our PLA intervention, we were therefore particularly conscious that continuing the trial may have increased risks amongst vulnerable populations with non-communicable diseases. The status of the trial at the point of suspension is summarised in Table 1. Bangladesh subsequently entered into a nationwide government-declared lockdown from the 23rd of March to the 30th of May 2020, and restrictions on mass gatherings continued until the 1st of September 2020 [5]. The second serious COVID-19 wave began in March 2021, and lockdowns were again implemented between 5th April–21st April 2021 and 1st July–11th August 2021.

**Table 1:** Status of the D:Clare stepped-wedge trial at the point of COVID-19 field activity suspension on the 20th of March 2020

	Milestone	Status
Administration	Ethical approvals	Approvals received from University College London (07/11/22) and the Diabetic Association of Bangladesh (03/12/19)
	Trial registration	Registered on 31/10/19
	Community entry, consent and public randomisation.	Meeting held 16/01/20
Evaluation	Community census for development of sampling frame	Data collection completed 04/02/20
	Recruitment and training of survey field staff	Training completed 10/02/20
	Baseline cross-sectional survey (target sample=1320 across the 12 study clusters)	Interrupted. 72% of the survey completed, with data gathered across all clusters, by 20/03/20. Follow-up data cleaning was conducted by phone.
Intervention	Recruitment and training of PLA community group intervention facilitators and supervisors	Completed
	Formation of PLA community groups in 6 clusters	Completed

This short article summarises the changes to our original trial design, in line with the CONSERVE 2021 Statement recommendations that trials impacted by extenuating circumstances should report on modifications [6]. We detail the considerations and rationale for these changes, which may be of relevance to other randomised controlled trials underway in dynamic contexts.

## Change in trial design

**Fig. 1:**
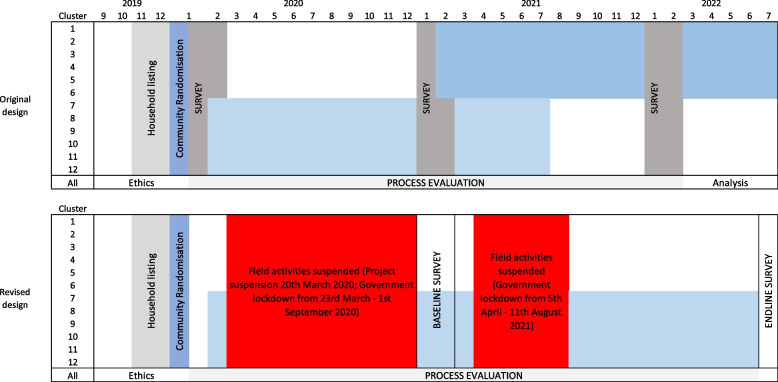
Planned D:Clare project timeline and COVID-19 interruptions

As a result of the changed risk landscape and the delays introduced by the COVID-19 pandemic, we decided to change from a stepped-wedge (SW-RCT) to a wait-list parallel arm cluster RCT (cRCT) with baseline data. Conceptually, our wait-list design is a parallel arm cRCT but with a commitment to implement the intervention to control clusters at the end of the trial evaluation. As detailed in Table 2, this differs from our stepped-wedge trial design in terms of the timing of roll-out of the intervention across all clusters, timing of cross-sectional data collection for evaluation, and in terms of how clusters are exposed over time, i.e. the allocated exposure (intervention or control) does not change during the trial evaluation. Our original SW design had two steps and was planned to take 30 months, with cross-sectional surveys done at months 1, 12 and 24 of intervention implementation (Fig. 1) [1]. The SW design should be resilient to temporal changes within a population, and so our original approach remained valid. However, the interruption of activities and the nature of the COVID-19 pandemic meant this design was no longer the most efficient and appropriate to meet project goals, and we presented alternative options to our Trial Steering Committee for consideration. We also engaged with community and government stakeholders to check that the proposed adaptions would be acceptable. We had four key reasons for eventually agreeing to change designs.

**Table 2:** Summary of key changes from original stepped-wedge design to parallel arm wait-list trial

**Trial component**	**Original stepped-wedge design**	**Parallel arm waitlist design**
**Intervention implementation**	Delivery to all clusters in two steps. 50% of clusters to receive the intervention from month 1 and the remaining 50% to receive the intervention from month 12.	Delivered to all clusters in two steps. 50% of clusters (i.e. intervention arm) to receive the intervention from month 1 and the remaining 50% (i.e. control arm) to receive the intervention after the trial end at approx. month 30.
	Up to 216 PLA community groups meeting on a monthly basis to progress through a schedule of 18 meetings over approx. 18 months.	Up to 216 PLA community groups meeting twice per month to progress through a schedule of a minimum of 13 meetings over a period of approx. 30 months.
	Total duration of implementation across all 12 clusters to be approx. 24 months.	Total duration of implementation across all 12 clusters to be approx. 30 months (including periods of ‘lockdown’ where intervention was paused.
**Timing of survey data collection**	At baseline (month 1), month 12, and month 24.	At baseline (month 1) and post intervention implementation in intervention clusters (approx. month 30).
**Cluster exposure over time**	Depends on timing - all clusters contribute data to both control and intervention exposure.	No change over time, i.e. intervention arm contributes to intervention exposure only, control arm contributes to control only.
**Total duration of project**	36 months	54 months

### Equipoise

The D:Clare PLA intervention was shown to be effective in reducing both the 2-year cumulative incidence and prevalence of T2DM in a rural Bangladesh population during the D-Magic trial [7]. This was part of the justification for us using an SW-RCT originally, as evidence of population benefit existed, and our aim was to determine effectiveness at scale in a similar but new population. However, with the considerable change in context and the potential need to adapt the intervention components and delivery, the lack of equipoise around the PLA intervention we had previously argued was less clear. Specifically, our intervention encourages groups to meet, encourages participation from those with T2DM and NCDs and encourages collective action. In a context where COVID-19 preventive measures focused on restricting inter-household interactions, we hypothesised that PLA’s mechanism of action may be affected.

**Fig. 2:**
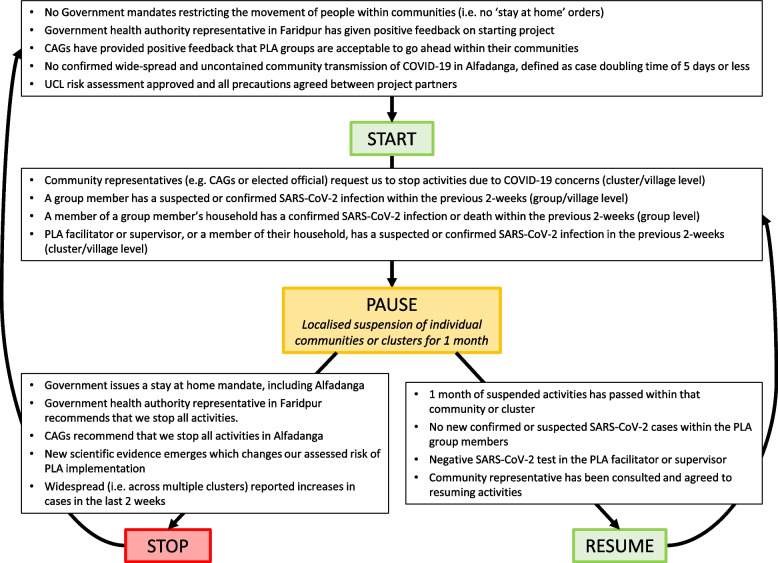
D:Clare trial stop, pause and start rules for COVID-19 adaptation

Further, if COVID-19 cases were not being diagnosed in this community setting, then group meetings had the potential to cause harm. However, by the end of 2020, there was evidence that outdoor environments posed a lower risk of transmission than indoor, crowded spaces, especially if this can be combined with the use of face masks, hand hygiene and physical distancing. Given the potential for our intervention to improve T2DM management (a key risk for poor COVID-19 outcomes), the ability to deliver in a way that would reduce transmission, and the inclusion of new stop/start rules (Fig. 2), we felt this risk could be sufficiently mitigated. We therefore decided we met the criteria for equipoise around the intervention needed to do a parallel arm cRCT.

### Temporal bias in exposure and outcomes

We also hypothesised that health literacy, care-seeking, dietary and physical activity behaviours, and the epidemiology of diabetes could be vastly different after lockdown restrictions were lifted — and therefore from our baseline survey. This in itself should not invalidate the SW-RCT design, but could make the interpretation and communication of the intervention impact on primary and secondary outcomes more complicated. The timing of intervention delivery relative to lockdown and social distancing measures was also likely to have an important influence on the uptake, delivery and effectiveness of the intervention. This may result in variable intervention effects between the two steps of SW implementation, which could be assessed through process evaluation, but again would complicate interpretation.

### Loss of power

Our power calculation was based on achieving at least an 80% response in the first cross-sectional survey. However, we only achieved 72% recruitment at the time of interruption and saw variation in rates between clusters (49–89%). In order to then ensure the SW-RCT was sufficiently powered, we would have had to increase the sample size of all the subsequent surveys. Switching to a parallel arm trial which uses both a new baseline and endline data (assuming an autocorrelation of 0.4), we could achieve 78% power for a 30% reduction in the primary outcome and considered this a feasible alternative. The change to the number and timing of surveys and the inclusion of baseline data in outcome evaluation are notable changes to our original protocol (Table 2).

### Time and funding

Finally, there was a very practical issue that we no longer had enough time to complete the SW-RCT design within the overall 36-month funded project period, using our 12-month staggered two-step design. By switching to a parallel cRCT we could complete the effectiveness evaluation within the funded project timeline, however, recognising that the parallel arm design would determine the intervention impact on a smaller population scale than we had originally intended. We then planned to source project extensions and explore the reallocation of resources to ensure that at a minimum the intervention could be delivered in control clusters as was promised to communities, but without incurring ongoing concurrent process, economic and impact evaluation costs.

## Protocol updates

We made changes to three key areas of the trial protocol: study design, intervention and sample size; no amendments were made to the trial procedures for population eligibility, sampling, randomisation, blinding, data collection, or analysis of the primary or secondary outcomes. A list of registered trial protocol amendments in our ISRCTN record is summarised in Table 3.

**Table 3:** Summary of registered trial protocol revisions in the D:Clare ISRCTN record

17/11/2021	1. Publication reference added. 2. The individual participant data (IPD) sharing statement has been updated.
17/12/2020	1. Ethics approval details added. 2. The study design was changed from ‘Stepped-wedge cluster randomized trial’ to a ‘Cluster randomized controlled trial’, with scale-up to control clusters after trial completion (‘wait-list’). 3. The interventions and primary and secondary outcome measures were updated. 4. The target number of participants measured across the baseline and endline surveys was changed from ‘12 clusters; 440 individuals per cluster’ to ‘12 clusters; 211 individuals per cluster’. 5. The recruitment start date was changed from 07/12/2019 to 04/01/2020.
06/03/2020	1. Ethics approval and secondary outcome measures updated.

We also set out COVID-19 standard operating procedures, with new stop-start rules (Fig. 2), and a COVID-19 safety protocol for staff and study participants, and consulted with a Data Monitoring and Safety Board on these infection prevention measures. For the intervention, we made the following modifications to incorporate COVID-19 measures: holding two meetings per village per month to allow for smaller groups but with the same coverage; inclusion of COVID-19 health information; re-organised meeting content to be delivered over a minimum of 13 instead of the planned 18 meetings (Table 2).

## Current trial status

As of 22/04/2022: We completed a new baseline survey on 25/02/2021, with a response rate of 1,392 from 1,584 (87.9%) sampled participants, which forms the parallel arm cRCT baseline data. A total of 213 PLA groups have been formed in 6 of the 12 study clusters, and have completed 11 of a minimum of 13 planned meetings. The endline survey will be completed between August and October 2022.

Protocol version 3.0 (16/06/2021)

## Data Availability

Data required to support the protocol can be supplied on request.

## Abbreviations

D:Clare: Diabetes: Community-Led Awareness, Response and Evaluation
NCD: Non-communicable disease
PLA: Participatory learning and action
RCT: Randomised controlled trial
SW-RCT: Stepped-wedge randomised controlled trial
T2DM: Type 2 diabetes mellitus
